# Tumor lymphangiogenesis index reveals the immune landscape and immunotherapy response in lung adenocarcinoma

**DOI:** 10.3389/fimmu.2024.1354339

**Published:** 2024-04-04

**Authors:** Weichang Yang, Zhijian Wu, Shanshan Cai, Zhouhua Li, Wenjun Wang, Juan Wu, Hongdan Luo, Xiaoqun Ye

**Affiliations:** ^1^ Department of Respiratory and Critical Care Medicine, The Second Affiliated Hospital of Nanchang University, Jiangxi Medical College, Nanchang University, Nanchang, Jiangxi, China; ^2^ Jiangxi Key Laboratory of Molecular Medicine, Nanchang, Jiangxi, China; ^3^ Department of Cardiology, The Second Affiliated Hospital of Nanchang University, Jiangxi Medical College, Nanchang University, Nanchang, Jiangxi, China

**Keywords:** lymphangiogenesis, lung adenocarcinoma, immune cell infiltration, prognosis, immunotherapy

## Abstract

**Background:**

Lymphangiogenesis (LYM) has an important role in tumor progression and is strongly associated with tumor metastasis. However, the clinical application of LYM has not progressed as expected. The potential value of LYM needs to be further developed in lung adenocarcinoma (LUAD) patients.

**Methods:**

The Sequencing data and clinical characteristics of LUAD patients were downloaded from The Cancer Genome Atlas and GEO databases. Multiple machine learning algorithms were used to screen feature genes and develop the LYM index. Immune cell infiltration, immune checkpoint expression, Tumor Immune Dysfunction and Exclusion (TIDE) algorithm and drug sensitivity analysis were used to explore the correlation of LYM index with immune profile and anti-tumor therapy.

**Results:**

We screened four lymphangiogenic feature genes (PECAM1, TIMP1, CXCL5 and PDGFB) to construct LYM index based on multiple machine learning algorithms. We divided LUAD patients into the high LYM index group and the low LYM index group based on the median LYM index. LYM index is a risk factor for the prognosis of LUAD patients. In addition, there was a significant difference in immune profile between high LYM index and low LYM index groups. LUAD patients in the low LYM index group seemed to benefit more from immunotherapy based on the results of TIDE algorithm.

**Conclusion:**

Overall, we confirmed that the LYM index is a prognostic risk factor and a valuable predictor of immunotherapy response in LUAD patients, which provides new evidence for the potential application of LYM.

## 1 Introduction

Lung cancer remains one of the major cancers threatening human health. A study reported approximately 2.2 million new cases of lung cancer and 1.8 million lung cancer deaths in 2020 (1). Unfortunately, the incidence of lung cancer continues to increase annually, and current treatments do not significantly improve the prognosis of lung cancer patients (2). Lung adenocarcinoma (LUAD) is the most common pathological type of lung cancer (3). LUAD is a highly aggressive and metastatic type of cancer (4). Therefore, elucidating the underlying mechanism of LUAD is highly important for its prevention and treatment.

Metastasis is a major cause of poor prognosis in tumor patients, and the lymphatic system is a common route for tumor metastasis (5). Lymphangiogenesis (LYM) is a key step in the entry of tumor cells into the lymphatic system (6). It has been reported that tumors promote LYM and further metastasis by secreting Tumor-Derived Decreted Factors (TDSFs), such as Vascular Endothelial Growth Factor C (VEGFC) (7). Tumor LYM is considered a key target for preventing tumor progression. Recent studies have suggested that the LYM may be a critical part of the premetastatic niche, in which tumors secrete TDSFs via lymphatic channels to create conditions favorable for tumor metastasis (5). Wang et al. reported that Tumor-Associated Macrophages (TAMs) also secrete inflammatory factors to promote LYM, suggesting that tumor immune cells are also involved in the process of LYM (8). Almost all tumor immune cells, including dendritic cells (DCs), tumor-associated fibroblasts and lymphocytes, can secrete factors that promote LYM (9). In addition, the density of lymphatic vessels in tumor tissue is considered a risk factor for the prognosis of tumor patients (10). LYM plays a crucial role in tumor metastasis and is a potential target for tumor treatment (11). However, the potential role of the LYM in LUAD has not been fully elucidated.

In this study, we developed the LYM index based on multiple machine learning algorithms. We further analyzed the correlation of the LYM index with patient prognosis, immune profile and immunotherapy efficacy, which provided new evidence for the development of LYM in LUAD.

## 2 Materials and methods

### 2.1 Data acquiring

**Figure 1:**
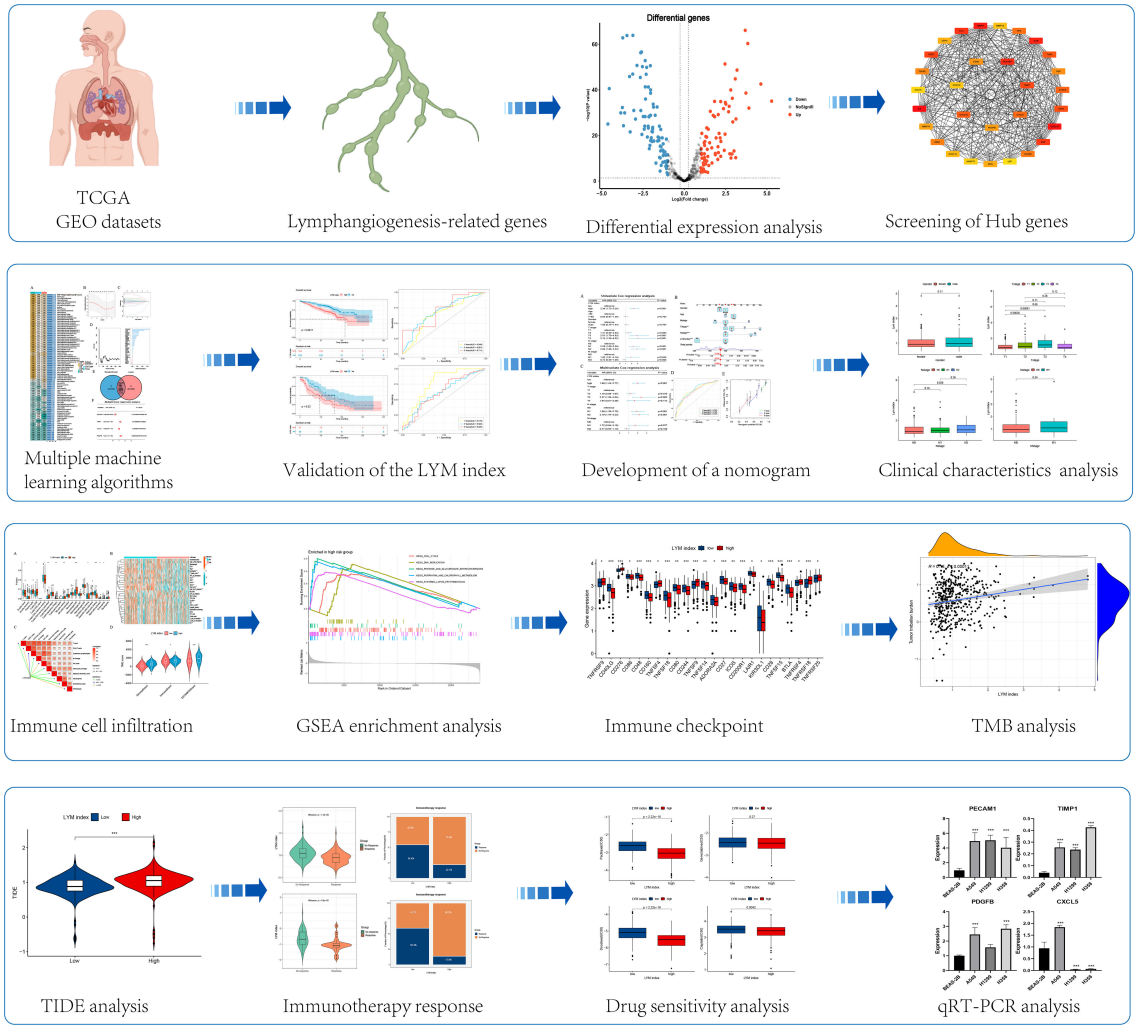
The flowchart of this study. *, p<0.05; **, p<0.01; ***, p<0.001.

We searched for “lymphangiogenesis” genes to obtain LYM-related genes from the GeneCards website (https://www.genecards.org/) and ultimately enrolled 466 LYM-related genes in this study; the specific process is shown in **Supplementary Figure S1**. The detailed list of LYM-related genes can be found in the **Supplementary Table S1**. The expression and survival data of LUAD patients were downloaded from The Cancer Genome Atlas (TCGA) and Gene Expression Omnibus (GEO) databases. We enrolled 1228 LUAD patients (TCGA: 460, GSE72094: 398, GSE36471: 115, GSE42127: 176, and GSE31210:79) in this study. The IMvigor210, GSE91061 and GSE78220 cohorts were obtained from the GEO database (https://www.ncbi.nlm.nih.gov/geo/). The immunohistochemistry (IHC) data were obtained from the Human Protein Atlas (HPA) database (https://www.proteinatlas.org/). The flow chart of this study is shown in **Figure 1**.

### 2.2 Differential expression analysis

A total of 466 LYM-related genes were used for differential expression analysis between LUAD and normal samples. A |log-fold change (logFC)| > 1 and adjusted p value < 0.05 were considered to indicate Differentially Expressed Genes (DEGs). The “limma” package was used in this study.

### 2.3 Enrichment analysis of DEGs

The “clusterProfiler” package was used to perform Gene Ontology (GO) and Kyoto Encyclopedia of Genes and Genomes (KEGG) enrichment analyses of the DEGs. The GO enrichment analyses included Biological Process (BP), Molecular Function (MF), and Cellular Component (CC) enrichment. The thresholds for the q value and p value were set at 0.05.

### 2.4 Screening hub genes based on DEGs

The STRING website (https://string-db.org/) was used to construct Protein-Protein Interactions (PPI) based on the DEGs, removing disconnected nodes in the network. Cytoscape software was used to screen the hub genes. The “CytoHubba” plugin was used to calculate the MCC values, and all the genes were sorted based on the MCC values.

### 2.5 Development and validation of LYM index

We screened the characterized genes to construct the LYM index based on multiple algorithms. LASSO regression analysis was used to avoid covariance and overfitting of the Hub genes. The “glmnet” package was used to perform the Lasso analysis. The “randomForestSRC” package was used to perform a Random Forest Survival (RFS) analysis to select feature genes based on the value of “importance”. Subsequently, multivariate Cox regression analysis was used to calculate the LYM index based on the “survival” package. The TCGA, GSE72094, and GSE36471 datasets were combined into a merged dataset after debatching effects were performed. The “sva” package was used for debatching effects. All patients were divided into two groups according to the median LYM index: high-LYM index and low-LYM index. The LYM index was further validated in the GSE42127 and GSE31210 datasets.

### 2.6 Construction of a nomogram

The “rms” package was used to construct the nomogram based on the LYM index, age, sex, T stage, N stage and M stage. The Receiver Operating Characteristic (ROC) curve was used to evaluate the areas under the curve (AUC) of the nomogram based on the “timeroc” package. A calibration curve was used to check the consistency of the nomogram model.

### 2.7 Gene set enrichment analysis

Gene set enrichment analysis (GSEA) was performed to compare high-LYM-index and low-LYM-index LUAD patients. The “c2.cp.kegg.v7.4.symbols” and “c5.go.v7.4.symbols” files were downloaded from https://www.gsea-msigdb.org/gsea/index.jsp. The “clusterProfiler” package was used to perform this analysis, and the p value threshold was set to 0.05.

### 2.8 Immune cell infiltration and immune microenvironment analysis

We analyzed the correlation between the LYM index and immune cell infiltration based on multiple algorithms (CIBERSORT, ssgsea and MCPcounter). The Wilcoxon test was used to compare the tumor microenvironment (TME) between LUAD patients with a high LYM index and low LYM index.

### 2.9 Immunotherapy response analysis

The Wilcoxon test was used to analyze the correlation of the LYM index with common immune checkpoint genes. The TIDE algorithm was used to analyze the correlation between the LYM index and immunotherapy efficacy. Tumor mutational burden (TMB) analysis was performed between LUAD patients with a high LYM index and low LYM index. The “maftools” package was used to perform this analysis. The IMvigor210, GSE91061 and GSE78220 cohorts were used to assess the correlation between LYM and immunotherapy response. The “pRRophetic” package was used to explore the value of the LYM index in predicting the efficacy of common antitumor drugs (vinblastine, cisplatin, paclitaxel, gemcitabine, docetaxel, and sorafenib) in LUAD patients.

### 2.10 Cell-line culture and quantitative real-time PCR

The BEAS-2B, A549, H1299 and H358 cell lines were obtained from the Jiangxi Key Laboratory of Molecular Medicine (1 Minde Road, Nanchang, Jiangxi Province). The cell culture conditions were as follows: DMEM (Solarbio, 11995) + 10% FBS (Solarbio, S9020) at 37°C and 5% CO_2_. The steps of quantitative real-time PCR (qRT-PCR) were as follows: Total RNA was isolated from the cells using Trizol reagent (Trizol Reagent, Ambion, China), and the extracted total RNA was reverse transcribed to cDNA using PrimeScriptTM RT reagent Kit with cDNA Eraser (TaKaRa) reagent, and stained using TB Green Premix Ex TaqTM II (TaKaRa) for staining, and the expression levels of the mRNAs of the 4 genes were detected according to the intensity of the fluorescent signals. Glyceraldehyde-3-phosphate dehydrogenase (GAPDH) RNA expression level was used as an endogenous control. Three experiments were performed for each sample, and the results were used to calculate the expression values of the 4 genes according to Equation 2-ΔΔCt. The primer sequences are shown in **Supplementary Table S2**.

### 2.11 Statistical analysis

R software (4.3.1) was used to perform all the statistical analyses in this study. The Wilcoxon test was used to compare differences between non-parametric variables. The chi-square test was used to analyze differences between categorical variables. The Spearman correlation analysis used to analyze the correlation between two continuous variables that are not normally distributed. The Kaplan-Meier analysis was used to analyze survival outcomes of categorical variables. A p value < 0.05 was considered to indicate a statistically significant difference.

## 3 Results

### 3.1 Differential gene expression analysis and enrichment analysis

**Figure 2:**
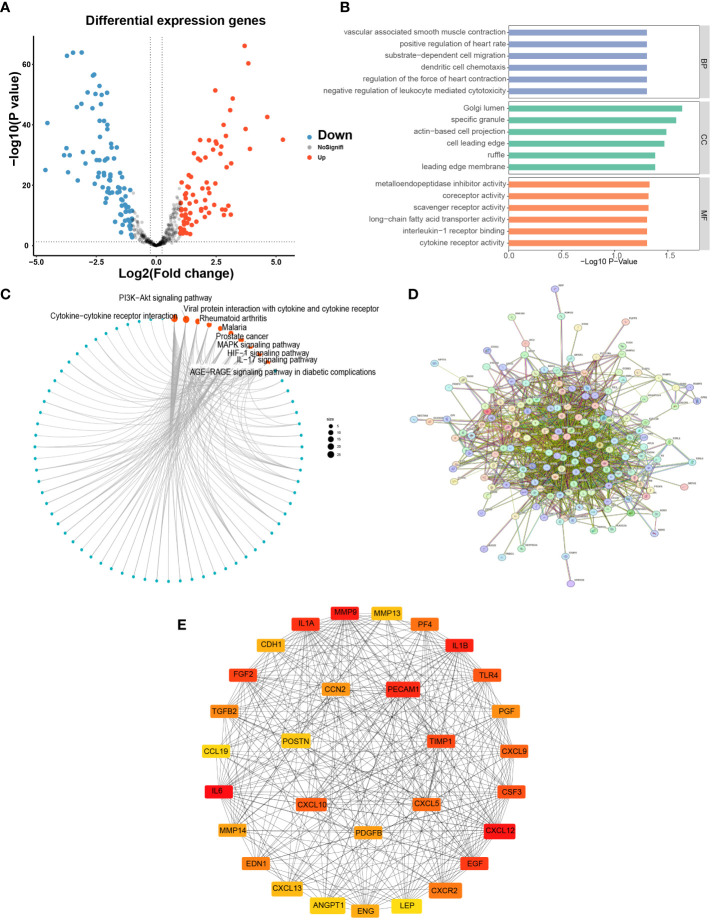
Differential expression analysis and enrichment of LYM-related genes. **(A)** Differential expression analysis of LYM-related genes. **(B)** GO enrichment analysis of DEGs. **(C)** KEGG enrichment analysis of DEGs. **(D)** PPI network construction of DEGs. **(E)** Screening top 30 Hub genes based on DEGs. DEGs, differentially expressed genes; GO, Gene Ontology; KEGG, Kyoto Encyclopedia of Genes and Genomes; LYM, lymphangiogenesis.

A total of 170 DEGs were obtained based on limma analysis, including 79 upregulated and 91 downregulated genes (**Figure 2A**). We analyzed 170 DEGs for GO and KEGG enrichment, we confirmed that LYM-related genes were enriched in Golgi lumen based on the results of CC (**Figure 2B**), and a previous study found that Golgi organization protein 1 promotes tumor LYM in oral squamous cell carcinoma (12), and these results suggested that the region where LYM exerts its function is associated with Golgi. The results of BP showed that these functions were involved in the metastasis of LUAD cells, for example, vascular-associated smooth muscle is regulated by the tumor suppressor protein p53, and cell migration is involved in tumor LYM (13, 14). The results of CC suggested that LYM was associated with metalloendopeptidase inhibitor, and blocking MMP inhibited lymphangiogenesis and lymph node metastasis (15), suggesting that the MMP family was widely involved in tumor LYM. In addition, further KEGG analysis suggested that PI3K−Akt, MAPK, HIF-1 and IL-17 signaling pathways were significantly enriched (**Figure 2C**). The hypoxic microenvironment provided favorable conditions for tumor LYM through activation of HIF-1 (16), activation of Akt has been reported to promote tumor LYM with metastasis (17), preventing the abnormal activation of MAPK significantly reduced the density of LYM (18) and IL-17 promoted LYM by facilitating VEGFC secretion (19). These results suggested that tumor LYM was a complex process regulated by multiple signaling pathways, providing new directions for the mechanism of LYM.

### 3.2 Screening of hub genes

To obtain key genes associated with LYM, 170 DEGs were imported into the STRING website to construct the PPI network (**Figure 2D**), and the disconnected genes in the network were removed. In addition, we imported the results of STRING into Cytoscape software to screen for hub genes and ultimately included the top 30 hub genes for the next analysis based on the sorting of MCC values (**Figure 2E**).

### 3.3 Constructing the LYM index based on machine learning

**Figure 3:**
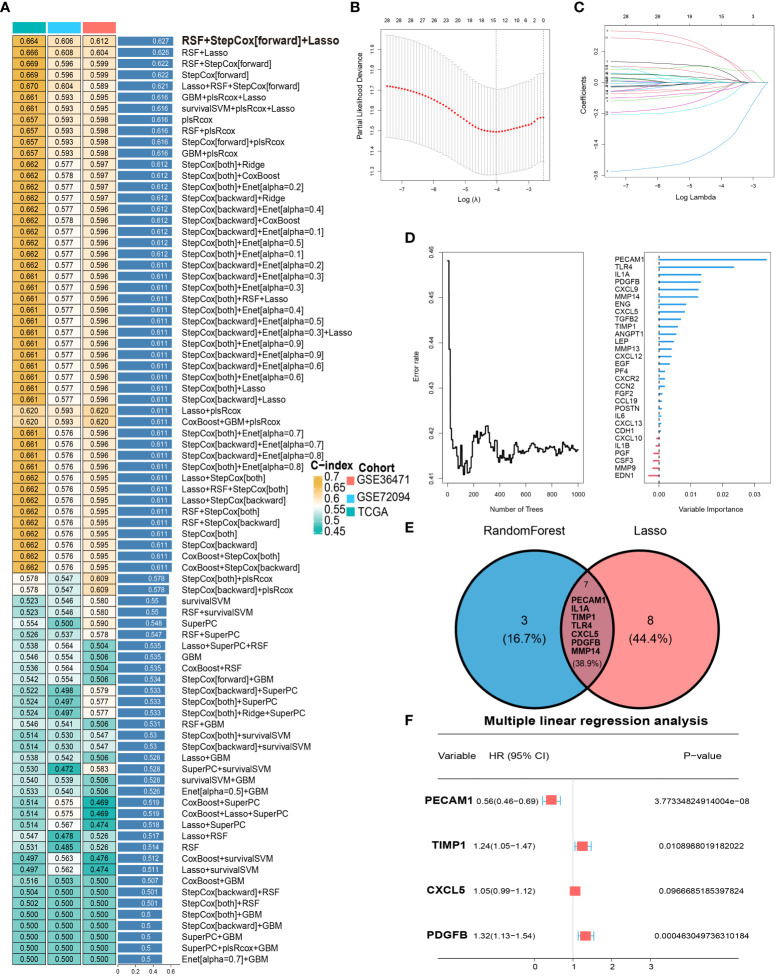
Development of LYM index. **(A)** Combination of multiple machine learning algorithms to calculate the C-index based on top 30 Hub genes. **(B)** LASSO coefficient profiles of 30 Hub genes. **(C)** Cross-validation of tuning parameter selection in the LASSO Cox regression. **(D)** The randomForestSRC of Hub genes. **(E)** Intersecting genes of Lasso and randomForestSRC. **(F)** The multiple linear Cox regression analysis of intersecting genes. The statistical analyses were performed using LASSO Cox regression, random forest analysis and multiple linear Cox regression.

To obtain the optimal C-index, multiple machine learning algorithms are combined to construct the LYM index. The RSF, Stepcox and lasso algorithms were used to screen feature genes via combination with multiple machine learning algorithms (**Figure 3A**). Therefore, we screened feature genes among the 30 Hub genes based on the RSF, Lasso and Stepcox algorithms. The results of the LASSO regression analysis showed that 15 genes (CXCL12, IL1B, PECAM1, IL1A, FGF2, TIMP1, CSF3, TLR4, CXCL5, PGF, PDGFB, MMP14, CDH1, CXCL13 and POSTN) were screened based on one thousandfold cross-validation (**Figures 3B, C**). We selected the top 10 genes (PECAM1, TLR4, IL1A, PDGFB, CXCL9, MMP14, ENG, CXCL5, TGFB2 and TIMP1) according to their importance based on the RSF results (**Figure 3D**). Seven genes (PECAM1, IL1A, TIMP1, TLR4, CXCL5, PDGFB and MMP14) were selected for the next step of multiple linear stepwise regression analysis based on the results of the Venn diagram (**Figure 3E**). PECAM1, TIMP1, CXCL5 and PDGFB were ultimately screened as feature genes and used to construct the LYM index (**Figure 3F**). LYM index= (PECAM1* -0.574960084211323) + (TIMP1* 0.215620339063194) + (CXCL5*0.0509418110154522) + (PDGFB* 0.275676188893711).

### 3.4 Validation of the LYM index

**Figure 4:**
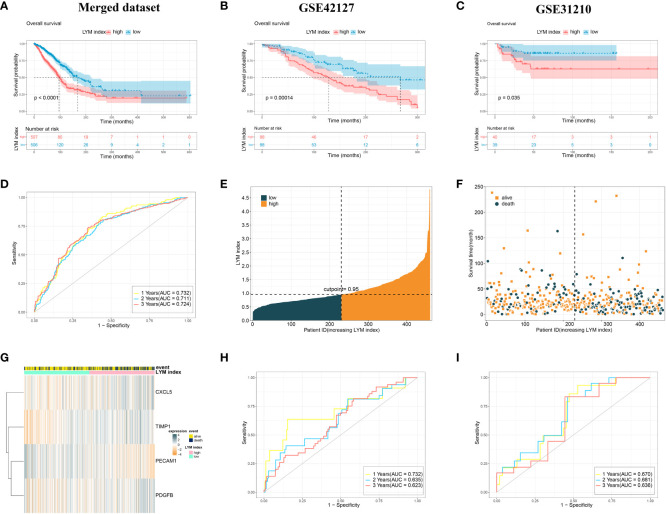
Validation of the LYM index. **(A)** The KM analysis of LYM index in merged dataset. **(B)** The KM analysis of LYM index in GSE42127. **(C)** The KM analysis of LYM index in GSE31210. **(D)** The AUCs at 1-, 2-, and 3-year of LYM index in merged dataset. **(E-G)** Distributions of LYM index and survival status of LUAD patients. **(H)** The AUCs at 1-, 2-, and 3-year of LYM index in GSE42127. **(I)** The AUCs at 1-, 2-, and 3-year of LYM index in GSE31210. LYM, lymphangiogenesis; KM, Kaplan-Meier; AUCs, areas under the curve. The statistical analyses were performed using KM analysis and ROC analysis.

We divided all LUAD patients into high-LYM-index and low-LYM-index groups based on the median LYM index (**Table 1**). The different batches were corrected to be at the same level, with significant differences before and after correction (**Supplementary Figures S2A, B**). Kaplan–Meier (KM) analysis confirmed that the high-LYM-index group had a worse prognosis than the low-LYM-index group (**Figure 4A**), GSE42127 (**Figure 4B**) and GSE31210 (**Figure 4C**), suggesting that the LYM index is a risk factor for LUAD patients. Progression-Free Survival (PFS) and Disease-Free Survival (DFS) are indicators for assessing the response of tumor patients to antitumor drugs. We analyzed the relationships between the LYM index and PFS and DFS, and the results showed that patients with a low LYM index had better PFS (**Supplementary Figure S2C**) and DFS (**Supplementary Figure S2D**).

**Table 1:** The clinical characteristics of lung adenocarcinoma patients in the high LYM index group and low LYM index group.

Group	High LYM index group (n=230)	Low LYM index group (n=230)	P-value
Age			p=0.001
>=60	n=159	n=189	
<60	n=71	n=41	
Gender			p=0.110
Male	n=114	n=98	
female	n=116	n=132	
T-stage			p=0.004
T1	n=64	n=96	
T2	n=127	n=112	
T3	n=30	n=14	
T4	n=9	n=8	
N-stage			p=0.025
N0	n=139	n=167	
N1	n=48	n=37	
N2	n=41	n=26	
N3	n=2	n=0	
M-stage			p=0.730
M0	n=154	n=152	
M1	n=12	n=9	
Mx	n=64	n=69	

LYM, lymphangiogenesis.

The ROC curve showed that the AUCs for 1-, 2-, and 3-year survival according to the LYM index were 0.732, 0.711 and 0.724, respectively, in the merged dataset (**Figure 4D**). Internal validation of the LYM index was performed on the merged database (**Figures 4E–G**). To assess the differentiation of the LYM index, we calculated the C-index of the LYM index. The results showed that the C-index was 0.696 (0.627-0.758). In addition, we validated the LYM index in the GSE42127 and GSE31210 cohorts. The AUCs for 1-, 2-, and 3-year survival according to the LYM index were 0.711, 0.635 and 0.623, respectively, in GSE42127 (**Figure 4H**) and 0.670, 0.681 and 0.636, respectively, in GSE31210 (**Figure 4I**). Based on the results of the C-index and AUC of the LYM index, we confirmed that the LYM index was a valuable indicator of the prognosis in LUAD patients. The AUC values were not high in the externally validated dataset (<0.7), which may be due to the small sample size and missing follow-up data. These results suggested that the LYM index is a robust indicator of the prognosis in LUAD patients.

### 3.5 Correlation of LYM index and vascular endothelial growth factor family

The Vascular Endothelial Growth Factor (VEGF) family plays an important role in the process of LYM in tumors (20), therefore clarifying the relationship between LYM index and VEGF helps to explain the authenticity of LYM index. We analyzed the correlation between the LYM index and VEGF family expression. The results showed that the LYM index was positively correlated with VEGFA (r=0.21, p<0.05) (**Supplementary Figure S3A**), VEGFB (r=0.076, p=0.1) (**Supplementary Figure S3B**) and VEGFC (r=0.17, p<0.05) (**Supplementary Figure S3C**), while it was negatively correlated with VEGFD (r=-0.49, p<0.05) (**Supplementary Figure 3D**). These results suggested that the expression of VEGF family was correlated with LYM index. We further analyzed the correlation of the expression of the 4 genes with the VEGF family. The results showed that PECAM1, TIMP1, CXCL5 and PDGFB were positively correlated with the VEGF family (VEGFA/B/C/D and VEGFR1/2/3) (**Supplementary Figure S3E**), and these results were consistent with previous studies (21).

### 3.6 Development of a nomogram based on LYM index

**Figure 5:**
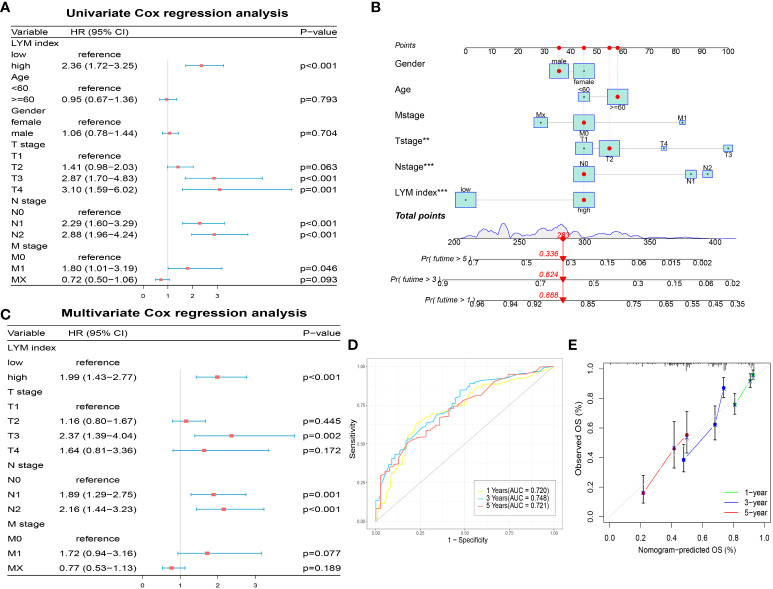
Development of a nomogram based on LYM index. **(A)** The univariate Cox regression analysis of LYM index and clinical features. **(B)** The nomogram based LYM index and clinical features. **(C)** The multivariate Cox regression analysis of LYM index and clinical features. **(D)** The AUCs at 1-, 3-, and 5-year of nomogram. **(E)** The calibration curve of nomogram. LYM, lymphangiogenesis; AUCs, areas under the curves. The statistical analyses were performed using univariate Cox regression analysis, multivariate Cox regression analysis and ROC analysis.

To explore the correlation between the LYM index and clinical characteristics, we analyzed the correlation between the LYM index and age, sex, and TNM stage in LUAD patients. The results showed that LYM index was higher in patients with age < 60 (**Supplementary Figure S4A**). LYM index differed between T1, T2 and T3 groups but not in T4 group (**Supplementary Figure S4B**), which may be due to small sample size in T4 group. For N-stage, LYM index differed between the N2 and N0 groups, while there was no significant difference in the N1 group (**Supplementary Figure S4C**). However, no significant differences were observed between LYM index and M-stage (**Supplementary Figure S4D**), Gender (**Supplementary Figure S4E**). To explore the prognostic value of the LYM index in LUAD patients, we performed univariate Cox regression analysis of the LYM index according to age, sex, T stage, N stage, and M stage. The results showed that the LYM index was associated with worse prognosis in LUAD patients (**Figure 5A**). The results of the multivariate Cox linear regression analysis showed that the LYM index was an independent risk factor for LUAD patients (**Figure 5C**). We developed a nomogram based on the LYM index, age, sex, T stage, N stage, and M stage to predict the survival probability of LUAD patients (**Figure 5B**). The AUCs at 1-, 3-, and 5-year of nomogram were 0.720, 0.748, and 0.721 (**Figure 5D**). The results of the calibration curve showed good accuracy of the nomogram (**Figure 5E**).

### 3.7 Correlations between LYM index and immune landscape

**Figure 6:**
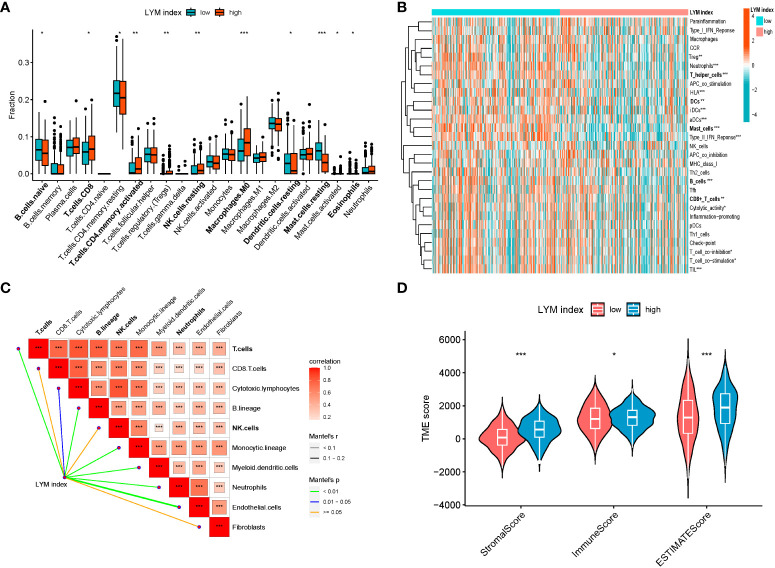
The correlations between LYM index and immune landscape. **(A)** Immune cell infiltration between two groups based on CIBERSORT. **(B)** Correlation of PMN index with immune cell infiltration based on ssGSEA. **(C)** Correlation of LYM index with immune cell infiltration based on MCPcounter. **(D)** The difference in tumor microenvironment between two groups. LYM, lymphangiogenesis. The statistical analyses were performed using Wilcoxon-Mann-Whitney test and Spearman analysis. *, p<0.05; **, p<0.01; ***, p<0.001.

To clarify the relationship between the LYM index and immune characteristics, we used multiple methods to analyze the correlation between the LYM index and immune cell infiltration. The results showed that fraction of CD8^+^ T cells, NK cells, DCs, macrophages, mast cells (MCs) and eosinophils were higher in the high-LYM-index group based on the CIBERSORT algorithm (**Figure 6A**). However, the fraction of MCs, neutrophils, T cells, DCs was higher in the low-LYM-index group based on the ssgsea algorithm (**Figure 6B**). We confirmed that LYM index was positively correlated with the fraction of T cells, endothelial cells, DCs, neutrophils and B cells based on the Mcpcounter algorithm (**Figure 6C**). These immune cells formed part of the TME and has been shown to contribute to tumor progression and immunotherapy response, the higher levels of tumor immune cells suggested better immunotherapy response (22). Considering the complexity of TME, we took these immune cells as a whole to assess TME in LUAD patients. TME analysis was composed of stromal score (the presence of tumor-associated stroma), immune score (the level of immune cell infiltration) and estimate score (comprehensive inference of tumor purity) (23). The results of the TME analysis suggested that immune score was higher in the low index group, while stromal score and estimate score were higher in the high index group (**Figure 6D**). High immune cell infiltration predicted a better response to immunotherapy, whereas the low-LYM-index group had a higher immune score, thus we concluded that the low-LYM-index group had a better response to immunotherapy.

### 3.8 GSEA analysis of LYM index

**Figure 7:**
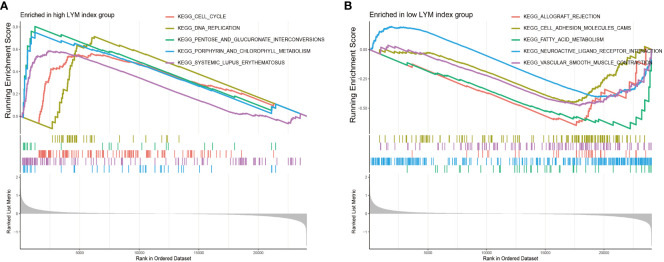
GSEA enrichment analyses between high LYM index group and low LYM index group. **(A)** GSEA enrichment analysis of high LYM index group. **(B)** GSEA enrichment analysis of low LYM index group. GSEA, Gene set enrichment analysis; LYM, lymphangiogenesis.

To clarify the difference between the high-LYM-index group and the low-LYM-index group, we performed GSEA on LUAD patients. The GSEA results indicated that cell cycle, DNA replication, pentose and glucuronate interconversions and porphyrin and chlorophyll metabolism were significantly enriched in the high-LYM-index group (**Figure 7A**); cell adhesion molecules, fatty acid metabolism and neuroative ligand-receptor interactions were significantly enriched in the low-LYM-index group (**Figure 7B**). YAP was reported to effectively block LYM through cell-cycle arrest and DNA replication inhibition (24), suggesting that cell cycle and DNA replication may be potential pathways in LUAD with high-LYM-index. Pentose and glucuronate interconversions and porphyrin and chlorophyll metabolism are common metabolic pathways, whose association with LYM has not been reported. However, these two metabolic pathways provide directions for the study of LYM in LUAD patients. The fatty acid metabolism was thought to inhibit LYM pathways, such as CPT1A, a key enzyme in fatty acid β-oxidation, inhibits LYM under pathological conditions (25), which may provide new evidence for the mechanism of LYM in LUAD with low-LYM-index. In addition, cell adhesion molecules and neuroative ligand-receptor interactions were involved in tumor progression (26–28), as indicated by the fact that CEACAM1, a cell adhesion molecule, was associated with tumor angiogenesis and LYM (29). Therefore, these pathways may reveal the potential mechanism involved in the difference between the high- and low-LYM-index groups.

### 3.9 The role of LYM index in immunotherapy

**Figure 8:**
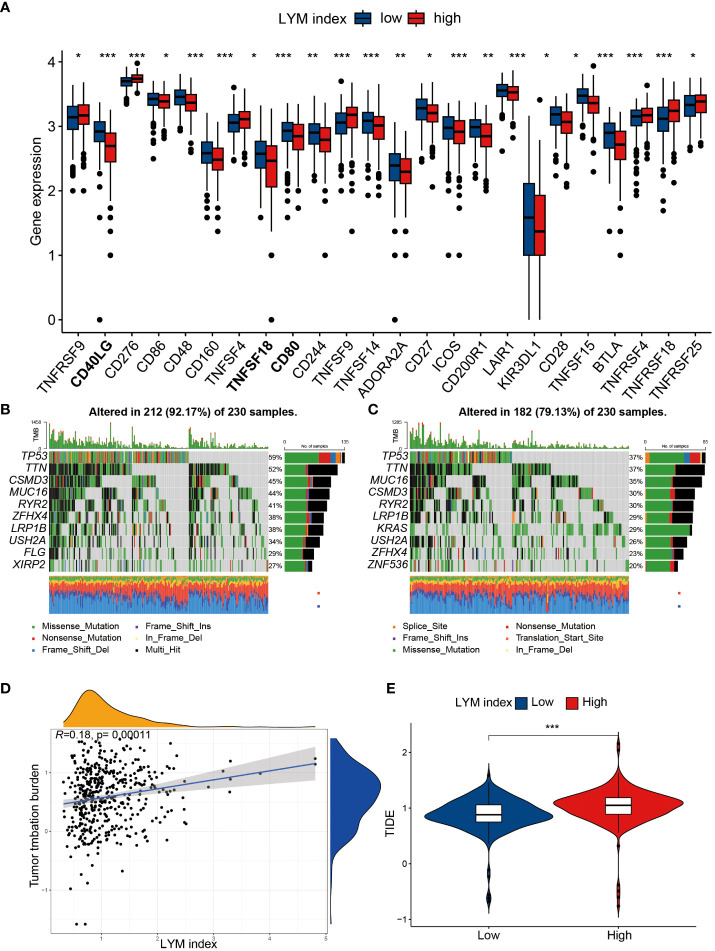
The correlation of LYM index with immunotherapy. **(A)** The differences in expression of immune checkpoints between high index group and low LYM index group. **(B)** TMB in the high LYM index group. **(C)** TMB in the low LYM index group. **(D)** The correlation between TMB score and LYM index. **(E)** The differences in TIDE scores between high and low LYM index groups. TMB, tumor mutational burden; TIDE, Tumor Immune Dysfunction and Exclusion; LYM, lymphangiogenesis. The statistical analyses were performed using Wilcoxon-Mann-Whitney test and Spearman analysis. *, p<0.05; **, p<0.01; ***, p<0.001.

To determine the value of the LYM index in guiding immunotherapy in LUAD patients, we analyzed the correlation of the LYM index with the TMB, immune checkpoint expression and TIDE score. We confirmed that most of the immune checkpoint genes (CD40LG, TNFSF18CD, and CD80) were highly expressed in the low-LYM-index group (**Figure 8A**). In addition, we directly analyzed the correlation between the LYM index and immune checkpoint-related genes and showed that the LYM index was significantly associated with the expression of most immune checkpoint genes (including TNFRSF4, TIGIT, IDO2, HAVCR2 and CD40LG) (**Supplementary Figure S5A**). We analyzed the correlation between the LYM index and PD-1/PDL-1 expression, and the results showed that there was no significant difference (**Supplementary Figure S5B**). TP53 was the most commonly mutated gene in both groups, whereas other genes, such as MUC16 and CSMD3, were differentially expressed (**Figures 8B, C**). In addition, the frequency of mutations in the high-LYM-index group was greater than that in the low-LYM-index group, suggesting that the LYM index may be associated with the frequency of mutations. We found that the LYM index was positively correlated with the TMB (r=0.18, p<0.05) (**Figure 8D**), although the correlation was not strong. The TIDE results confirmed that the patients in the low-LYM-index group had a lower TIDE score than did those in the high-LYM-index group (**Figure 8E**).

We further explored the correlation of the LYM index with immunotherapy response in the IMvigor210, GSE78220 and GSE91061 cohorts. The results showed that patients who responded to immunotherapy had a lower LYM index, and the proportion of patients who responded to immunotherapy was greater in the low-LYM-index group than in the high-LYM-index group in the IMvigor210 (**Supplementary Figures S6A, B**) and GSE91061 cohorts (**Supplementary Figures S6C, D**); moreover, no significant difference was observed in the GSE78220 cohort (**Supplementary Figures S6D, E**). These results suggested that the patients in the low-LYM-index group benefited more from immunotherapy, which is consistent with the TIDE algorithm. The above results suggested that the LYM index may be a potential marker for predicting immunotherapy response.

### 3.10 Drug sensitivity analysis

**Figure 9:**
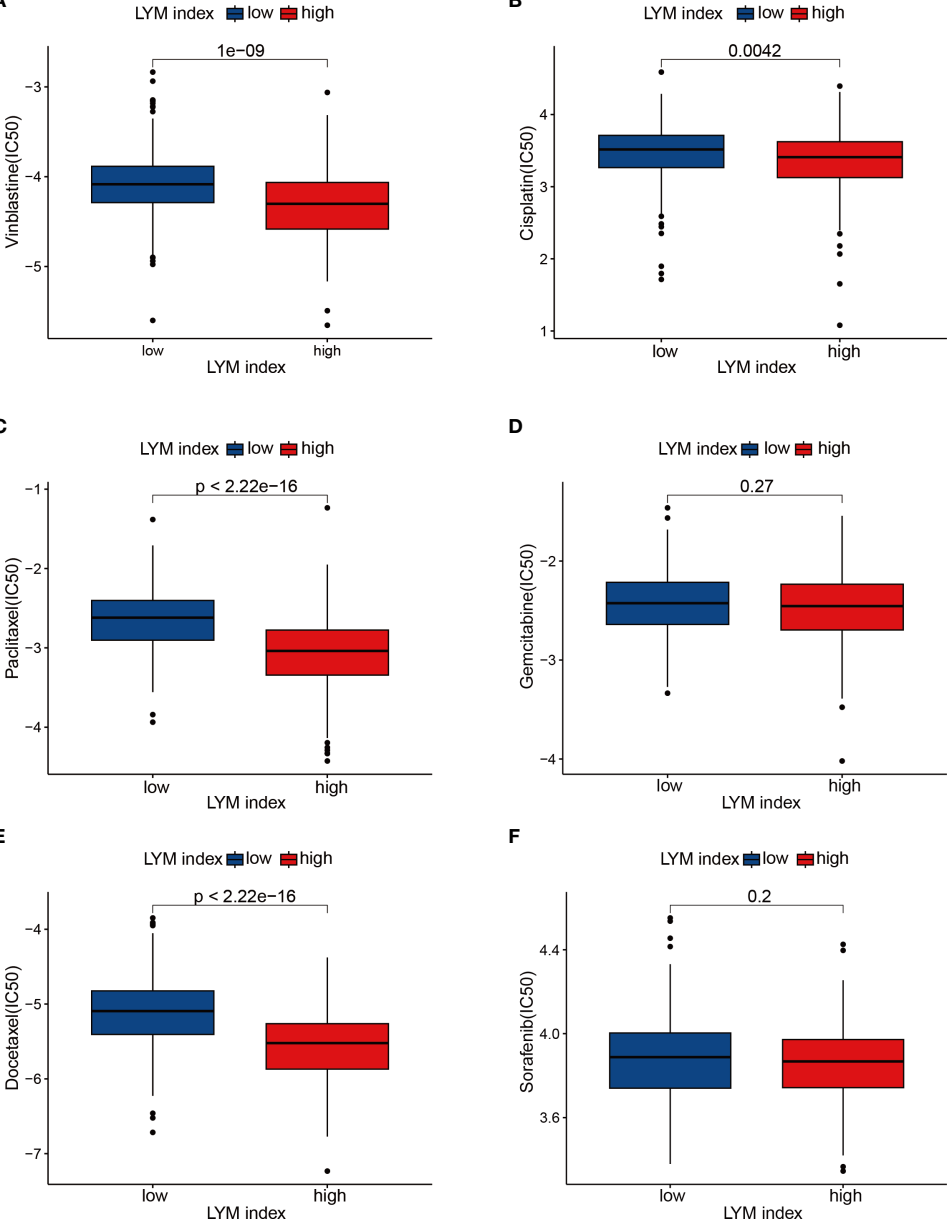
Drug sensitivity analyses. **(A)** Vinblastine, **(B)** Cisplatin, **(C)** Paclitaxel, **(D)** Gemcitabine, **(E)** Docetaxel, **(F)** Sorafenib. The statistical analysis was performed using Wilcoxon-Mann-Whitney test.

To determine the correlation between the LYM index in LUAD patients and sensitivity to common antitumor drugs, we performed a drug sensitivity analysis. The results of the drug sensitivity analysis confirmed that vinblastine (**Figure 9A**), cisplatin (**Figure 9B**), paclitaxel (**Figure 9C**), and docetaxel (**Figure 9E**) had lower IC50 values in the high-LYM-index group, while there were no significant differences between the high-index and low-LYM-index groups for gemcitabine (**Figure 9D**) or sorafenib (**Figure 9F**). The above results suggested that patients in the high-LYM-index group were more sensitive to common antitumor drugs.

### 3.11 Validation of the expression of featured genes

**Figure 10:**
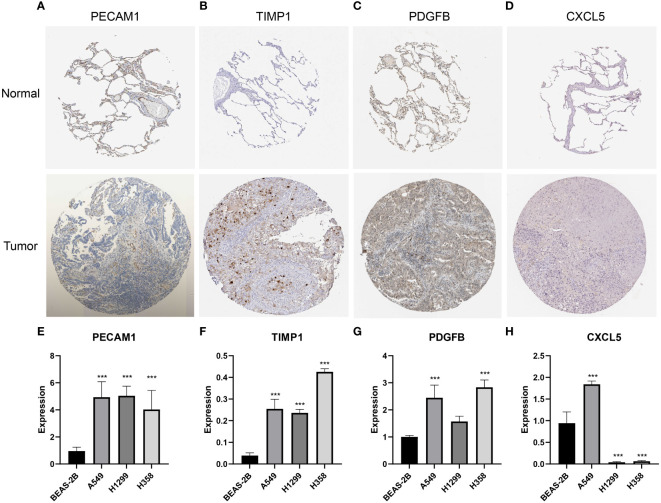
Validation of the expression of featured genes. **(A-D)** The immunohistochemistry of PECAM1 (n=3), TIMP1 (n=3), PDGFB (n=3) and CXCL5 (n=3). **(E–H)** The qRT-PCR analysis of PECAM1 (n=3), TIMP1 (n=3), PDGFB (n=3) and CXCL5 (n=3). The statistical analysis was performed using Wilcoxon-Mann-Whitney test. ***, p<0.001.

To validate the differences in the expression of the feature genes between LUAD tissues and normal tissues, we performed IHC analysis based on the HPA database and qRT-PCR analysis. The IHC results demonstrated that PECAM1 (**Figure 10A**), TIMP1 (**Figure 10B**) and PDGFB (**Figure 10C**) were highly expressed in LUAD tissues, and there was no significant difference in the expression of CXCL5 (**Figure 10D**) between LUAD tissues and normal tissues. The qRT-PCR results suggested that the expression of PECAM1 (**Figure 10E**), TIMP1 (**Figure 10F**) and PDGFB (**Figure 10G**) in LUAD cells (A549, H1299 and H358 cells) were higher than in BEAS-2B cells. The expression of CXCL5 was higher in A549 cells than in BEAS-2B cells; however, it was lower in H1299 and H358 cells than in BEAS-2B cells (**Figure 10H**). The differences in the expression of the 4 feature genes between lung cancer cells and normal lung epithelial cells helped to validate the feasibility of the LYM index.

## 4 Discussion

LYM is an important process in tumor metastasis, providing a shortcut for the spread of tumor cells (30). LYM is a potential target for blocking tumor metastasis; however, the clinical application of LYM is still limited (31). In this study, we developed an index for assessing the tumor LYM based on LUAD data. Furthermore, we confirmed the correlation of the LYM index with immune cell infiltration, patient prognosis, and immunotherapy response.

We developed the LYM index through multiple algorithms based on 4 genes (PECAM1, TIMP1, CXCL5 and PDGFB). PECAM1, also called CD31, is normally expressed in vascular endothelial cells and platelets and aggregates at cell junctions (32). We confirmed that PECAM1 was a protective factor for LYM. PECAM1 was shown to be associated with LYM in coronary artery disease, chronic venous disease during pregnancy and tumors (33–35). Simons et al. found that lymphatic endothelial cells (LECs) were impaired in PECAM1-deficient mice, suggesting that PECAM1 impairs LYM by affecting LECs development (36). TIMPs are important inhibitors of MMPs, and TIMP1 is essential for tumor cell proliferation and migration (37). TIMP1 promoted the invasive capacity of tumor cells and accelerated tumor progression, which appears to be distinct from the traditional function of MMP inhibitors (38, 39). We confirmed a positive correlation between TIMP1 expression and the LYM index, which supported the function of TIMP1 in promoting tumor progression. CXCL5, a chemokine, promoted tumor cell proliferation and metastasis through multiple pathways (40). Chakraborty et al. demonstrated that tumor-derived CXCL5 significantly stimulates tumor LYM and promotes tumor cell metastasis (41), which could explain the correlation between CXCL5 and LYM in our study. Tumor-secreted PDGFB stimulates tumor LYM and further promotes lymph node metastasis (42, 43), and PDGFB may be an effective target for inhibiting LYM (44), which could explain the association between PDGFB and the LYM index. We confirmed the correlation between VEGFA/C/D and LYM index, except for VEGFB. Previous studies demonstrated that VEGFA/C/D promotes tumor LYM and lymph node metastasis (45, 46); however VEGFB has not been reported. We also confirmed that 4 genes (PECAM1, TIMP1, CXCL5 and PDGFB) were correlated with the expression of the VEGF family, suggesting that these genes may work together with the VEGF family to promote tumor LYM. The correlation between VEGF and LYM index suggested that LYM index may truly reflect tumor LYM.

LUAD patients were divided into high-and low-LYM index groups based on the median value of the LYM index. LYM index was developed based on LYM-related genes. LYM is closely associated with LUAD progression and metastasis (47), and based on the role of LYM in LUAD, we concluded that metastasis, invasiveness, and malignancy were higher in the LUAD patients in the high LYM index group compared to the low LYM index group. We confirmed that the prognosis of LUAD patients with a low LYM index was better than that of patients with a high LYM index, suggesting that LYM was an independent risk factor for LUAD, which is consistent with the findings of previous studies (48–50). The survival probability of patients with lymph node metastasis is significantly reduced, and the presence of lymphatic vessels provides a convenient channel for identifying tumor lymph node metastasis (51). We also confirmed differences in the LYM index between the different N-stage groups, and the LYM index was significantly greater in patients with lymph node metastasis than in those without lymph node metastasis. However, the specific mechanism of LYM has not been determined.

Based on the results of 170 DEGs significantly enriched for functions and pathways, activation of Akt promote tumor LYM with metastasis (17), the hypoxic microenvironment created favorable conditions for tumor LYM (52), and IL-17 promoted LYM by facilitating VEGFC secretion (19). Additionally, the differences in pathways associated with the high- and low-LYM-index groups further revealed the pathways that may be involved in LYM based on GSEA. Abnormal cell cycle progression and DNA replication are characteristics of tumor proliferation (53, 54), and G1 cell cycle arrest is associated with LYM (55). We confirmed that the cell cycle and DNA replication were significantly enriched in the high-LYM-index group, suggesting that LYM in LUAD patients may be associated with these pathways. In addition, we confirmed that pentose and glucuronate interconversions and porphyrin and chlorophyll metabolism were significantly enriched in the high-index group, and fatty acid metabolism was significantly enriched in the low-index group. Pentose and porphyrin are common metabolic pathway molecules in tumor cells and play important roles in tumor progression (56, 57), but the relevance of these molecules to tumor LYM has not been reported. Cell adhesion molecules, neuroative ligand-receptor interactions and vascular smooth muscle contraction were significantly enriched in the low-index group. These pathways have been reported to promote tumor cell proliferation and migration (58), suggesting the potential mechanism for LYM in the low-index group. Overall, the identification of the pathways associated with the differential expression between the patients with a high index and low index provided new directions for identifying potential mechanisms of tumor LYM.

Tumor-infiltrating immune cells (TIICs) are important factors in tumor LYM. TAMs, neutrophils, MCs, B cells and DCs were reported to promote LYM through the secretion of growth factors (VEGFA/C/D) (59). The role of immature DCs is to present antigens to T cells, and mature dendritic cells can secrete certain factors to promote the immune response (60). Using a hormonal mouse model, Brüne et al. demonstrated that LYM was inhibited by S1PR1 deletion in TAMs (61), suggesting the involvement of TAMs in tumor LYM. We found that these cells were more abundant in the high-LYM-index group than in the low-LYM-index group, suggesting that tumor LYM may require additional immune cell infiltration. TIICs are a major component of the TME and help to explain the higher stromal scores and estimated stromal scores in the high-LYM-index group. Swartz et al. reported that tumor LYM enhanced immune responses by promoting T-cell infiltration (62), which suggested that a high LYM index may be associated with improved immunotherapy responses. Our study identified differences in the TME and TIICs between the high- and low-LYM-index groups, and these differences suggested that LYM may be induced not only by tumor cells but also by TIICs, which revealed the complexity of tumor LYM.

With the rise of tumor immunotherapy, the role of lymphatic in immunotherapy is gradually gaining attention. Recent studies have reported that tumor LYM enhances the immune response of tumors by stimulating T-cell activation and further enhancing the immune response (62). The TMB is considered a useful indicator for assessing the response to immunotherapy (63). Studies have reported that tumor patients with a high TMB produce more antigens and are more likely to induce an immune response from immune cells (64). We found that the LYM index was positively correlated with the TMB; however, the correlation coefficient was only 0.18, so further studies are needed to assess this correlation. We confirmed that the proportion of immune therapy response was greater in the low-LYM group than in the high-LYM group for PD-1/PDL-1 therapy based on the IMvigor210 and GSE91061 cohorts, although no correlation was observed between the LYM index and PD-1 expression. The TIDE algorithm is a tool used to predict immunotherapy response in tumor patients, and higher TIDE scores correlate with poorer immunotherapy efficacy (65). We found that the TIDE score was lower in the low-LYM-index group than in the high-LYM-index group, suggesting that patients in the low-LYM-index group benefited more from ICB therapy, which may explain the greater survival probability of patients in the low-LYM-index group. Overall, we confirmed that the LYM index is a valuable predictor of immunotherapy response in LUAD patients.

There are still several limitations of our study. First, we validated only the differences in the expression of LYM feature genes, and additional animal and clinical studies need to be performed to validate the potential value of the LYM index. Second, all the data for our study were obtained from public databases and a larger sample size is needed to assess the prognostic value of the LYM index.

## 5 Conclusion

We developed the LYM index based on multiple machine learning algorithms and demonstrated its prognostic value for LUAD patients. In addition, we further confirmed the correlation of the LYM index with the immune landscape and immunotherapy response in LUAD patients. Overall, the LYM index is a prognostic risk factor for LUAD patients and contributes to guiding immunotherapy in LUAD patients, providing new evidence for the potential application of the LYM index.

## Data availability statement

The datasets presented in this study can be found in online repositories. The names of the repository/repositories and accession number(s) can be found in the article/**Supplementary Material**.

## Author contributions

WY: Methodology, Validation, Visualization, Writing – original draft, Writing – review & editing. ZW: Conceptualization, Writing – review & editing. SC: Formal Analysis, Methodology, Software, Writing – review & editing. ZL: Methodology, Project administration, Writing – review & editing. WW: Conceptualization, Supervision, Validation, Writing – review & editing. JW: Data curation, Project administration, Validation, Writing – review & editing. HL: Data curation, Project administration, Validation, Writing – review & editing. XY: Supervision, Writing – review & editing.

## Supplementary material

Supplementary Figure S1 The flowchart for screening differentially expressed genes of lymphangiogenesis. Unrelated genes: genes that are not included in the TCGA database but are included in the Genecards database.

Supplementary Figure S2 The KM analysis of PFS and DFS with LYM index. **(A)** PCA plot of expression data before normalization. **(B)** PCA plot of expression data after normalization. **(C)** The KM analysis of PFS. **(D)** The KM analysis of DFS. PFS, progression free survival; DFS, disease free survival; KM, Kaplan-Meier; LYM, lymphangiogenesis. The statistical analysis was performed using KM analysis.

Supplementary Figure S3 The correlation of LYM index with VEGF family. **(A)** The correlation of LYM index with VEGFA. **(B)** The correlation of LYM index with VEGFB. **(C)** The correlation of LYM index with VEGFC. **(D)** The correlation of LYM index with VEGFD. **(E)** The correlation of 4genes with VEGF family. LYM, lymphangiogenesis. The statistical analysis was performed using Spearman analysis. *: p<0.05, **: p<0.01, ***: p<0.001.

Supplementary Figure S4 |The correlation of clinical characteristics with LYM index. **(A)** age, **(B)** T-stage, **(C)** N-stage, **(D)** gender, **(E)** M-stage. LYM, lymphangiogenesis. The statistical analysis was performed using Wilcoxon-Mann-Whitney test.

Supplementary Figure S5 The correlation of LYM index with immune checkpoint genes. **(A)** The correlation of LYM index with immune checkpoint genes. **(B)** The correlation of LYM index with PD-L1. LYM, lymphangiogenesis. The statistical analysis was performed using Spearman analysis. *: p<0.05, **: p<0.01, ***: p<0.001.

Supplementary Figure S6 The immunotherapy response analysis of LYM in the IMvigor210, GSE78220 and GSE91061 cohorts. **(A)** Distribution of LYM index between response and no-response in the IMvigor210 cohort. **(B)** Comparison of overall response rates between the high LYM index group and low LYM index group of the IMvigor210 cohort. **(C)** Distribution of LYM index between response and no-response in the GSE91061 cohort. **(D)** Comparison of overall response rates between the high LYM index group and low LYM index group of the GSE91061 cohort. **(E)** Distribution of LYM index between response and no-response in the GSE78220 cohort. **(F)** Comparison of overall response rates between the high LYM index group and low LYM index group of the GSE78220 cohort. LYM, lymphangiogenesis. The statistical analysis was performed using Wilcoxon-Mann-Whitney test.
