# Supplementary figures and images for: Tumor lymphangiogenesis index reveals the immune landscape and immunotherapy response in lung adenocarcinoma
Source: Front Immunol. 2024 Apr 4;15:1354339. doi: 10.3389/fimmu.2024.1354339 (PMC11024352; doi:10.3389/fimmu.2024.1354339)

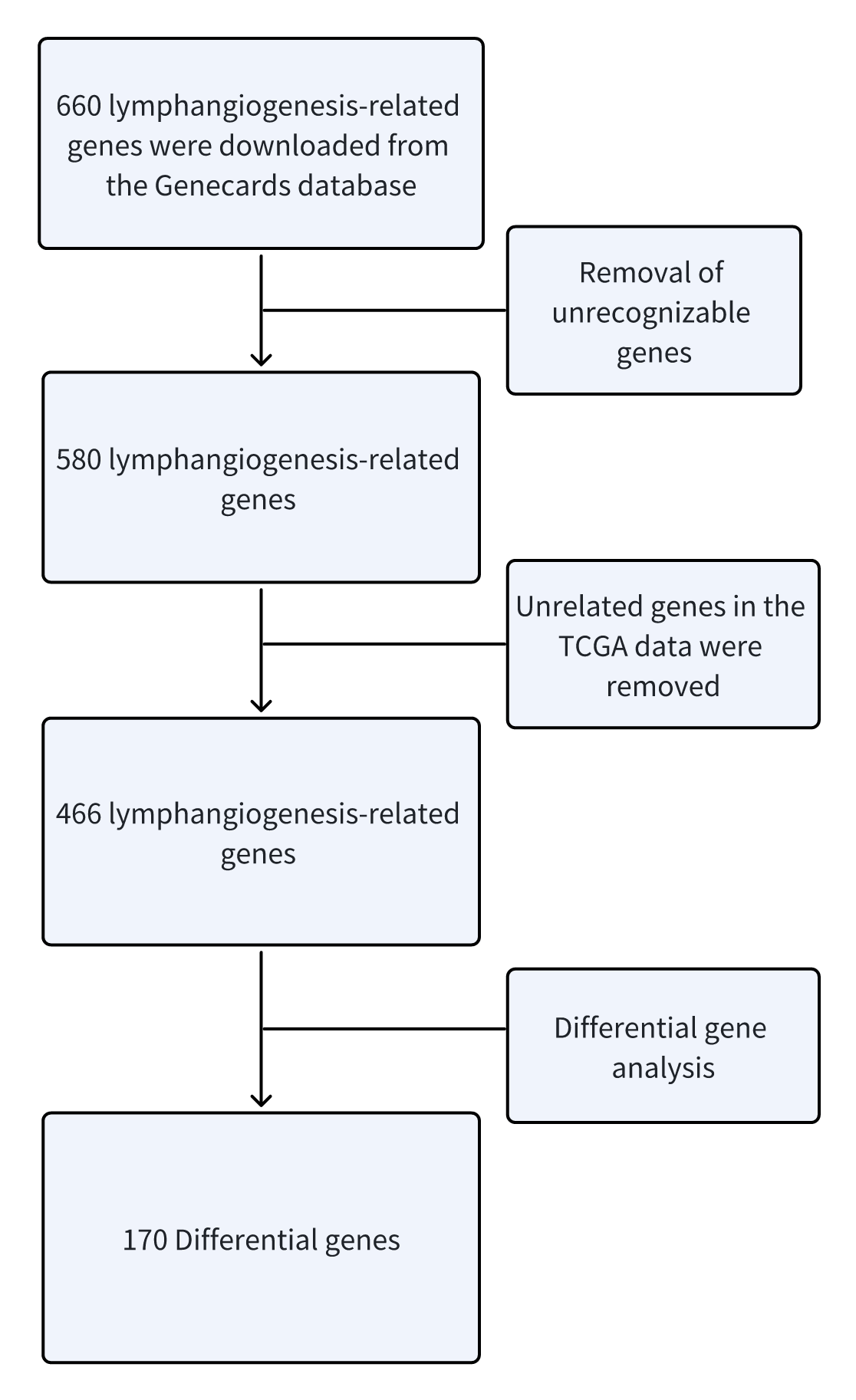

Supplement: Supplementary Figure S1 — The flowchart for screening differentially expressed genes of lymphangiogenesis. Unrelated genes: genes that are not included in the TCGA database but are included in the Genecards database. [file Image_1.tif]

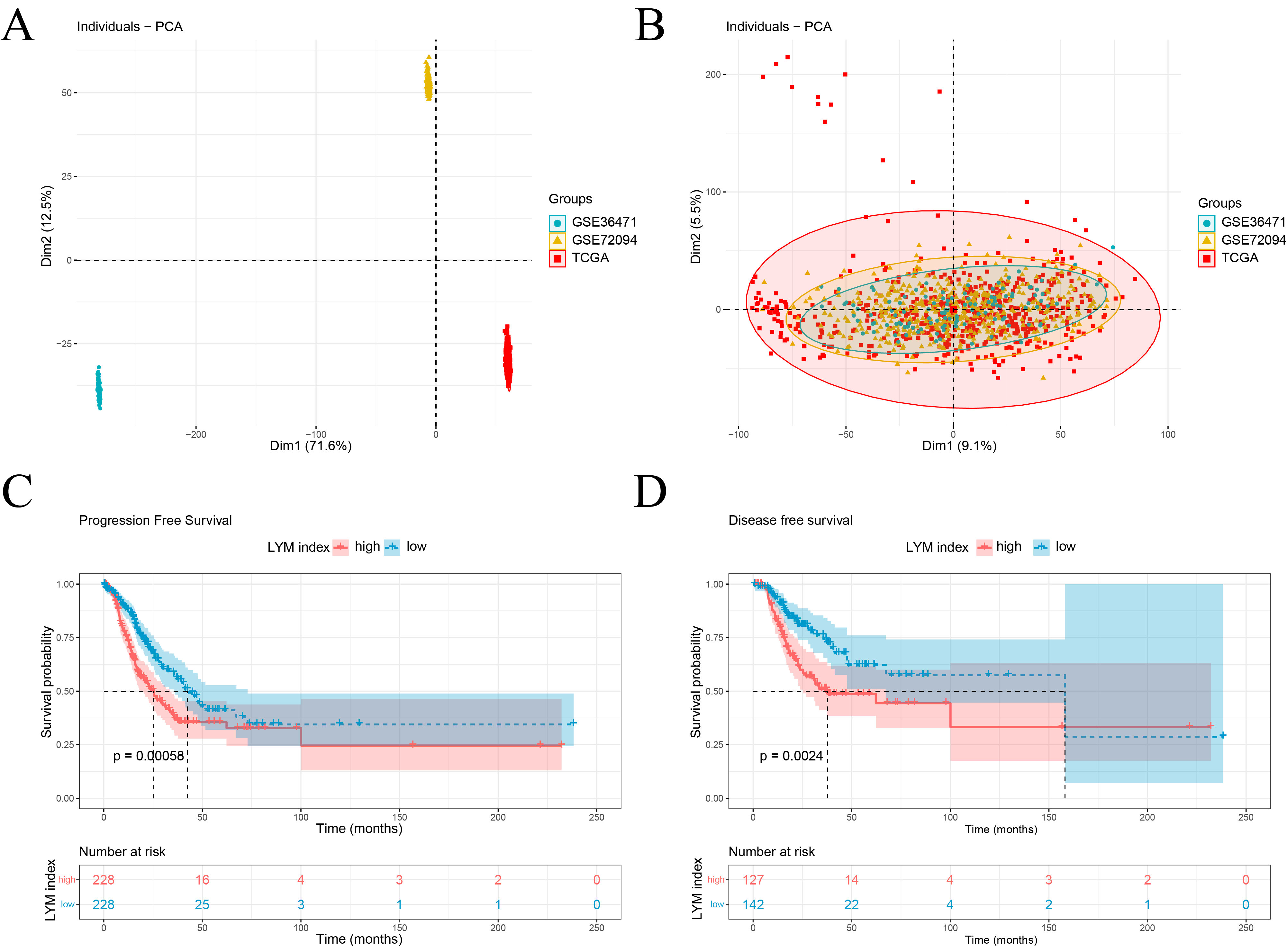

Supplement: Supplementary Figure S2 — The KM analysis of PFS and DFS with LYM index. (A) PCA plot of expression data before normalization. (B) PCA plot of expression data after normalization. (C) The KM analysis of PFS. (D) The KM analysis of DFS. PFS, progression free survival; DFS, disease free survival; KM, Kaplan-Meier; LYM, lymphangiogenesis. The statistical analysis was performed using KM analysis. [file Image_2.tif]

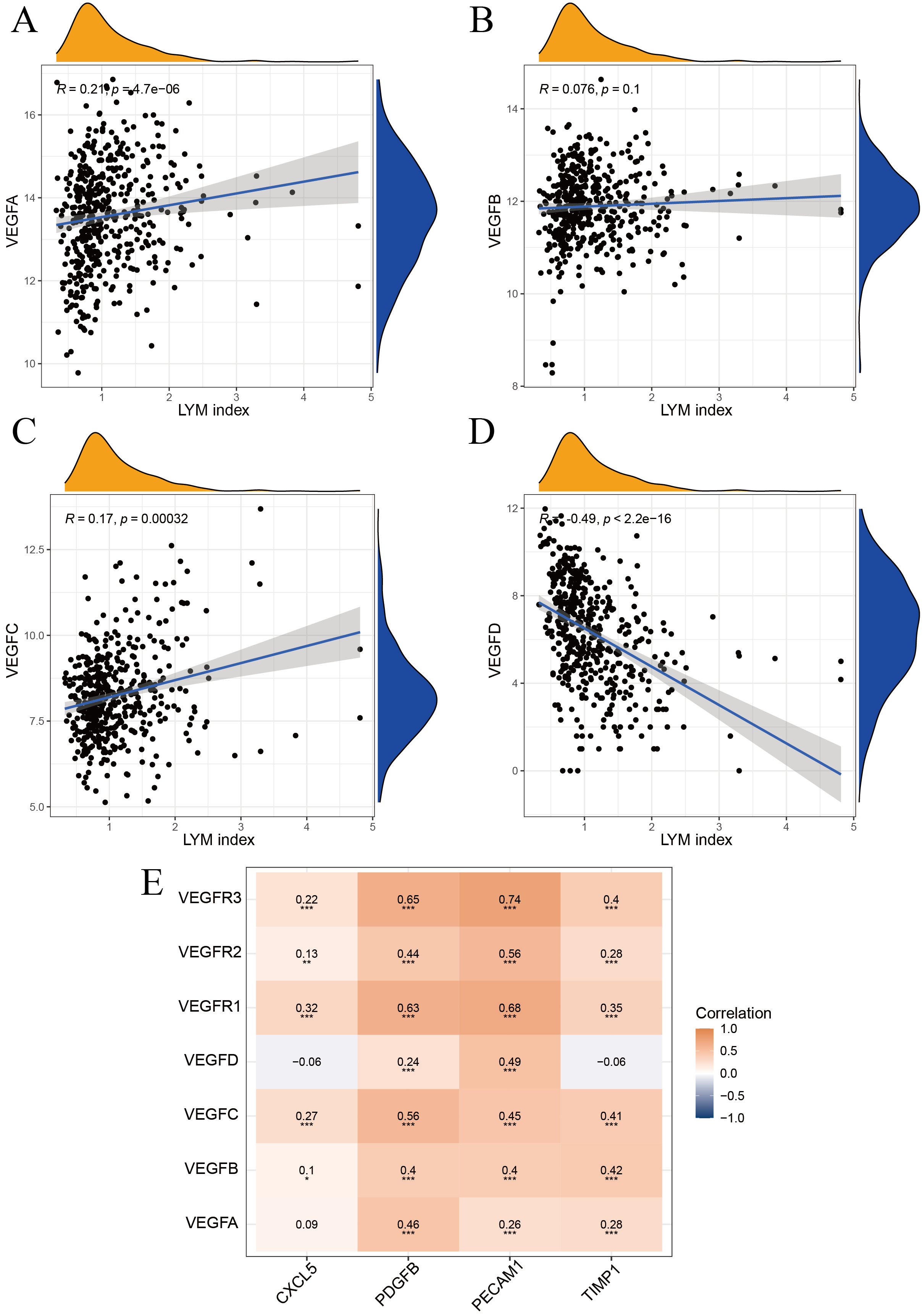

Supplement: Supplementary Figure S3 — The correlation of LYM index with VEGF family. (A) The correlation of LYM index with VEGFA. (B) The correlation of LYM index with VEGFB. (C) The correlation of LYM index with VEGFC. (D) The correlation of LYM index with VEGFD. (E) The correlation of 4genes with VEGF family. LYM, lymphangiogenesis. The statistical analysis was performed using Spearman analysis. *: p<0.05, **: p<0.01, ***: p<0.001. [file Image_3.tif]

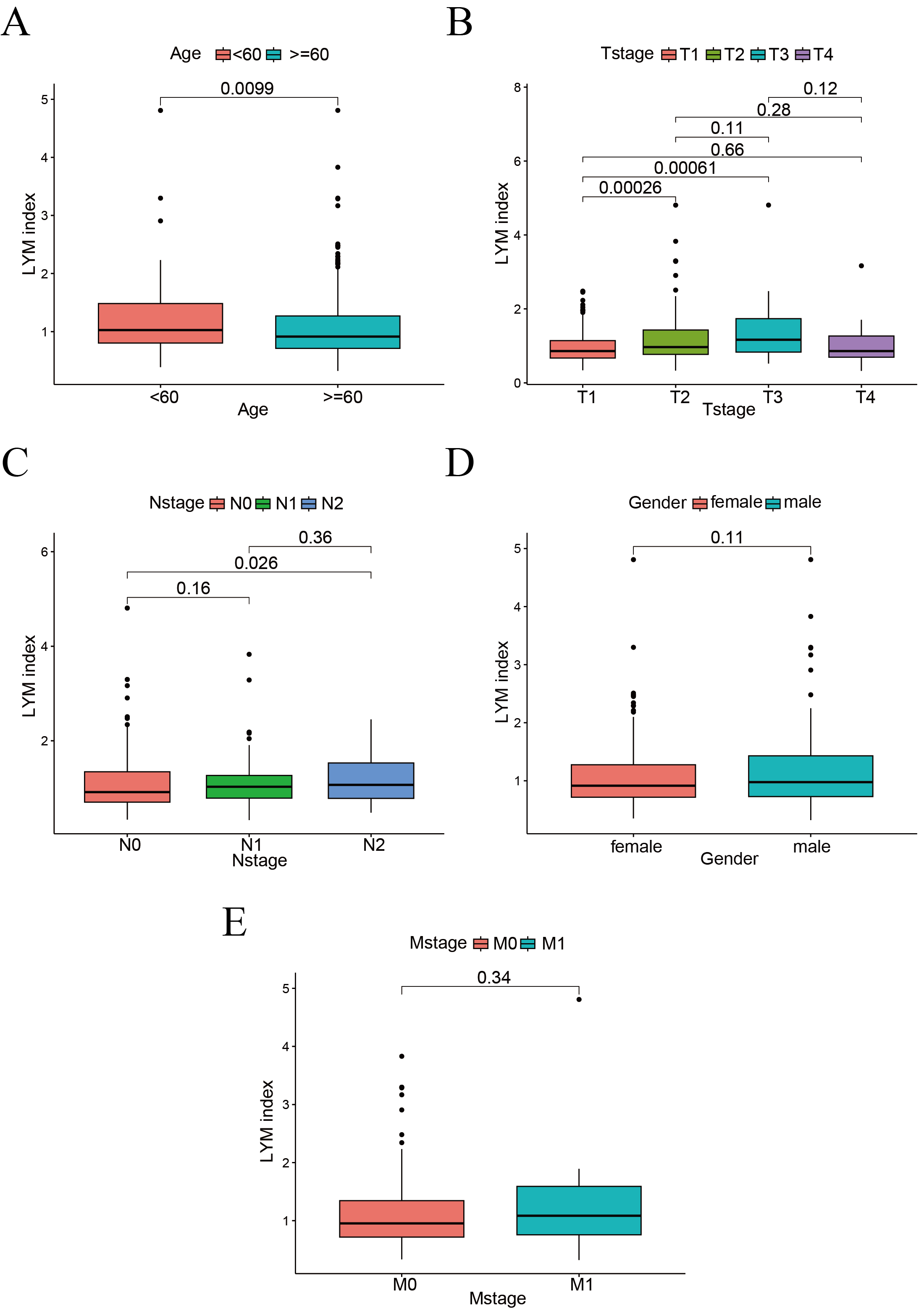

Supplement: Supplementary Figure S4 — |The correlation of clinical characteristics with LYM index. (A) age, (B) T-stage, (C) N-stage, (D) gender, (E) M-stage. LYM, lymphangiogenesis. The statistical analysis was performed using Wilcoxon-Mann-Whitney test. [file Image_4.tif]

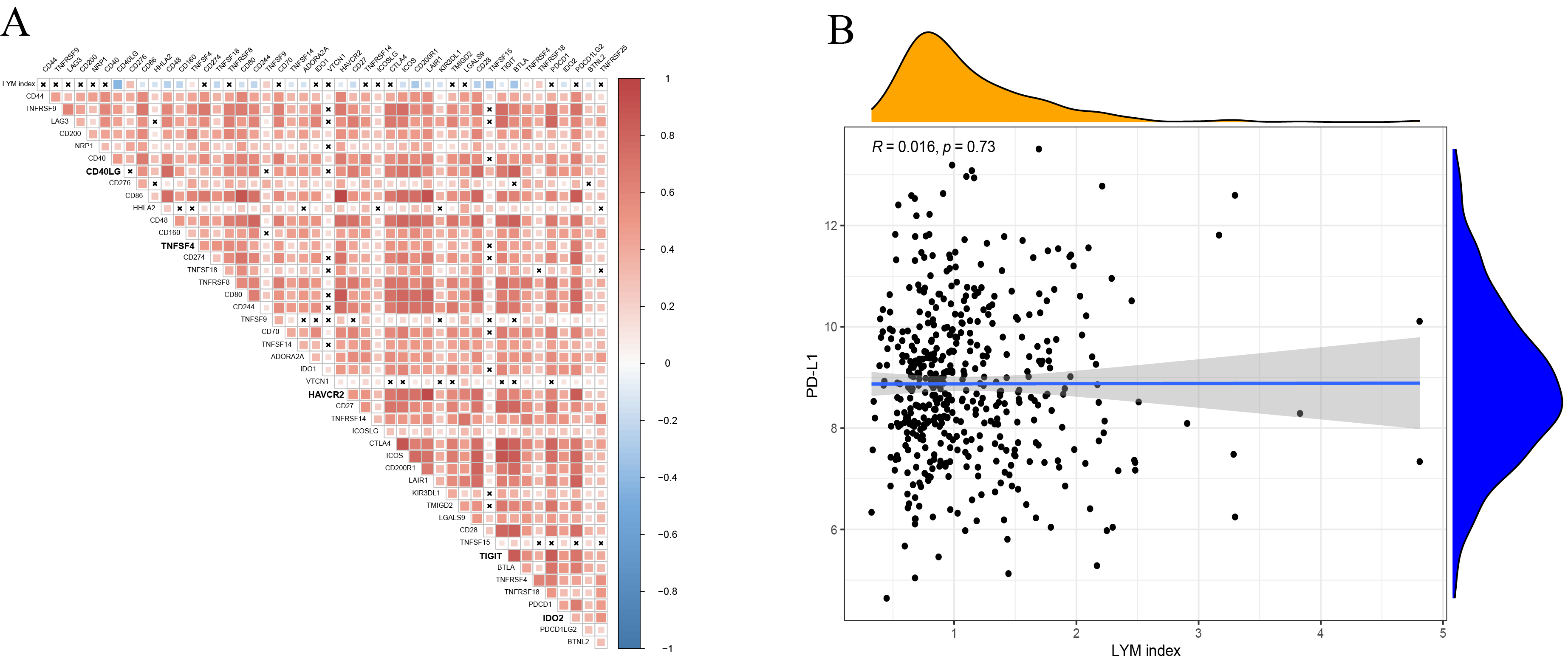

Supplement: Supplementary Figure S5 — The correlation of LYM index with immune checkpoint genes. (A) The correlation of LYM index with immune checkpoint genes. (B) The correlation of LYM index with PD-L1. LYM, lymphangiogenesis. The statistical analysis was performed using Spearman analysis. *: p<0.05, **: p<0.01, ***: p<0.001. [file Image_5.tif]

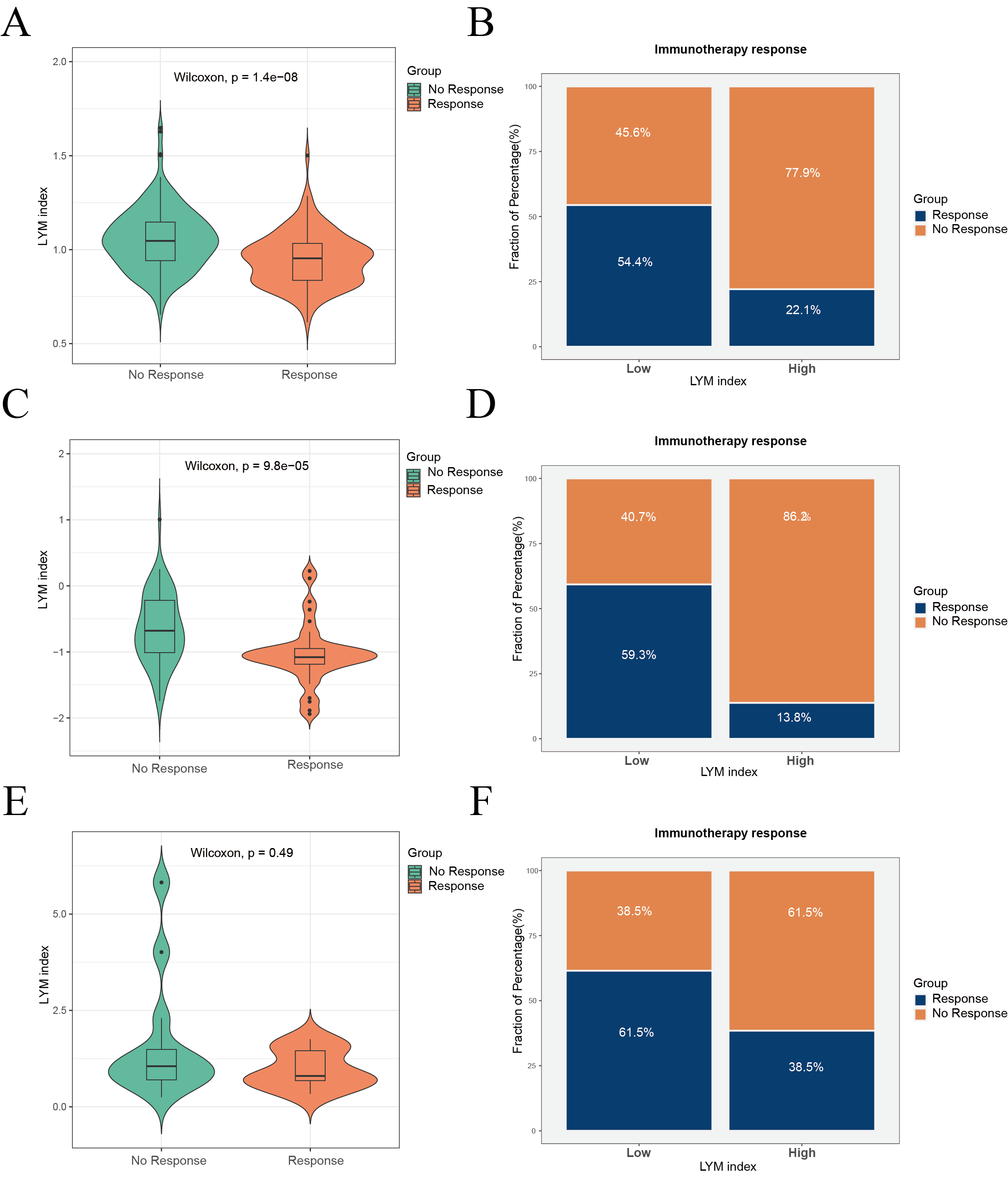

Supplement: Supplementary Figure S6 — The immunotherapy response analysis of LYM in the IMvigor210, GSE78220 and GSE91061 cohorts. (A) Distribution of LYM index between response and no-response in the IMvigor210 cohort. (B) Comparison of overall response rates between the high LYM index group and low LYM index group of the IMvigor210 cohort. (C) Distribution of LYM index between response and no-response in the GSE91061 cohort. (D) Comparison of overall response rates between the high LYM index group and low LYM index group of the GSE91061 cohort. (E) Distribution of LYM index between response and no-response in the GSE78220 cohort. (F) Comparison of overall response rates between the high LYM index group and low LYM index group of the GSE78220 cohort. LYM, lymphangiogenesis. The statistical analysis was performed using Wilcoxon-Mann-Whitney test. [file Image_6.tif]
